# A Review of Odd-Chain Fatty Acid Metabolism and the Role of Pentadecanoic Acid (C15:0) and Heptadecanoic Acid (C17:0) in Health and Disease

**DOI:** 10.3390/molecules20022425

**Published:** 2015-01-30

**Authors:** Benjamin Jenkins, James A. West, Albert Koulman

**Affiliations:** MRC HNR, Elsie Widdowson Laboratory, Fulbourn Road, Cambridge CB1 9NL, UK; E-Mails: Benjamin.Jenkins@mrc-hnr.cam.ac.uk (B.J.); James.West@mrc-hnr.cam.ac.uk (J.A.W.)

**Keywords:** odd chain fatty acids, pentadecanoic, heptadecanoic, biomarker, α-oxidation, dairy

## Abstract

The role of C17:0 and C15:0 in human health has recently been reinforced following a number of important biological and nutritional observations. Historically, odd chain saturated fatty acids (OCS-FAs) were used as internal standards in GC-MS methods of total fatty acids and LC-MS methods of intact lipids, as it was thought their concentrations were insignificant in humans. However, it has been thought that increased consumption of dairy products has an association with an increase in blood plasma OCS-FAs. However, there is currently no direct evidence but rather a casual association through epidemiology studies. Furthermore, a number of studies on cardiometabolic diseases have shown that plasma concentrations of OCS-FAs are associated with lower disease risk, although the mechanism responsible for this is debated. One possible mechanism for the endogenous production of OCS-FAs is α-oxidation, involving the activation, then hydroxylation of the α-carbon, followed by the removal of the terminal carboxyl group. Differentiation human adipocytes showed a distinct increase in the concentration of OCS-FAs, which was possibly caused through α-oxidation. Further evidence for an endogenous pathway, is in human plasma, where the ratio of C15:0 to C17:0 is approximately 1:2 which is contradictory to the expected levels of C15:0 to C17:0 roughly 2:1 as detected in dairy fat. We review the literature on the dietary consumption of OCS-FAs and their potential endogenous metabolism.

## 1. Introduction

The development of chromatographic technologies has enabled the study of lipid biochemistry and the role lipids play in the pathology of many diseases. There has been an ever increasing drive to improve the resolution and sensitivity of lipid analysis starting from thin layer chromatography several decades ago to ultra-performance liquid chromatography coupled to high resolution mass spectrometry. This has led to a considerable development in the understanding of lipids and their associations with disease, through disease etiology, biomarkers, treatment and prevention. To the present date, there have been over 150 different diseases connected with lipids, ranging from high blood pressure and artery plaques [1], obesity [2], type II diabetes [3], cancer [4] and neurological disorders [5].

Fatty acids are the basic building blocks of more complex lipids [6] and their composition in different lipid species are often used as a means for comparison within a lipid class when examining disease and physiological perturbations in lipid metabolism. It has been shown that saturated fatty acids [7] are associated with increased relative risks for diseases such as coronary heart disease, atherosclerosis, fatty liver disease, inflammatory diseases and Alzheimer’s disease. In contrast many unsaturated fatty acids including both mono-unsaturated and poly-unsaturated, have been associated with a reduced risk for each of the previously described disorders in certain studies [8]. Fatty acid chain length is also used for the diagnosis and prognosis of disease with respect to adrenoleukodystrophy, Refsum disease and Zellweger Syndrome where the propagation of very long chain fatty acids (>22 Carbon length chain [9]) is indicative of these disorders [10].

The majority of research into fatty acid metabolism has been conducted primarily on even chain fatty acids (carbon chain length of 2–26) as these represent >99% of the total fatty acid plasma concentration in humans [11,12]. However there is also a detectable amount of odd-chain fatty acids in human tissue. As a result of the low concentration there are only four significantly measureable odd chain fatty acids, which are C15:0, C17:0, C17:1 [13] and C23:0 [14]. C15:0 and C17:0; these have been gaining research interest within the scientific community as they have been found to be important as: (1) quantitative internal standards; (2) biomarkers for dietary food intake assessment; (3) biomarkers for coronary heart disease (CHD) risk and type II diabetes mellitus (T2D) risk (although the objective is not to provide a meta-analysis of odd chain saturated fatty acids (OCS-FAs) and disease risk); (4) evidence for theories of alternate endogenous metabolic pathways, where these are discussed hereafter. The purpose of this review is to address these points and highlight the importance of their inclusion into routine lipidomic analyses, as well as introduce areas that need further research.

## 2. Discussion

### 2.1. A Quantitative Internal Standard (Q-Int. Std.)

Since the early 1960s, it has been concluded that odd chain saturated fatty acids (OCS-FAs) are of little physiological significance [15,16,17] and that the only real difference with their more abundant counterparts, even chain fatty acids [12], is seen in the endpoint of metabolism where OCS-FAs result in propionyl CoA [17] as opposed to acetyl CoA [18]. Moreover, the OCS-FAs are present at apparently insignificant plasma concentrations [19] (<0.5% total plasma fatty acid concentration [20]) and the natural variation of concentrations within blood plasma ranging from 0%–1% (Table 1). 

Table 1(**A**,**B**) The relative concentrations of plasma fatty acid (12 to 24 number of carbons), within four lipid classes, showing the reported variation in the reporting of fatty acid profiles and the differences in the reported levels of OCS-FAs; NEFA—non-esterified fatty acids (free fatty acids), PL—Phospholipids, CE—Cholesterol esters, GL (TAGs)—Glycerolipids (Triacylglycerols). (A) Different studies showing the relative concentrations in plasma of fatty acids (12 to 24 carbons) with in different lipid classes; NEFA—non-esterified fatty acids (free fatty acids) [21,22,23,24,25], PL—Phospholipids [3,26,27,28,29]; (B) Different studies showing the relative concentrations in plasma of fatty acids (12 to 24 carbons) with in different lipid classes; CE—Cholesterol esters [3,25,30,31,32], GL (TAGs)—Glycerolipids (Triacylglycerols) [30,33,34,35,36].

**Table:** 

(A)	NEFA					PL					
Saturated Fatty Acid	*n* = 14, [21]	*n* = 15, [22]	*n* = 27, [23]	*n* = 200, [24]	*n* = *, [25]	*n* = 1224, [26]	*n* = 15164, [27]	*n* = 4930, [12]	*n* = 195, [28]	*n* = 178, [29]	*n* = 2657, [3]
12:0	*	0.65	0.31	0.02	0.33	0.07	*	*	*	*	*
12:1	*	*	0.065	*	*	*	*	*	*	*	*
13:0	*	*	*	*	*	*	*	*	*	*	*
14:0	3.5	2.96	1.93	0.74	2.79	0.69	0.36	*	0.27	0.32	*
14:1	*	*	0.23	0.04	*	*	*	*	*	*	*
**15:0**	*	**0.88**	**0.4**	**0.17**	**0.30**	**0.23**	**0.21**	*	**0.15**	**0.15**	*
15:1	*	*	*	*	*	*	*	*	*	*	*
16:0	35.2	35.38	26.66	22.78	29.39	30.54	29.93	*	25.34	26.3	25.3
16:1	2.7	2.01	5.5	2.64	6.77	0.95	*	*	0.65	0.79	0.63
16:2	*	*	0.05	*	*	*	*	*	*	*	*
**17:0**	*	**1.25**	**0.45**	**0.28**	**0.55**	*	**0.41**	*	**0.33**	**0.41**	*
17:1	*	0.3	0.38	*	0.47	*	*	*	*	*	*
17:2	*	*	0.009	*	*	*	*	*	*	*	*
18:0	40.7	31.57	7.82	6.76	10.18	13.11	14.05	*	14.1	11.6	13.2
18:1	8.7	*	40.37	22.45	36.99	10.44	*	*	12.31	9.7	8.66
18:2	9.2	7.53	9.96	*	7.00	20.91	*	*	23.09	27.3	22.1
18:3	*	0.64	1.39	0.49	0.53	0.30	*	*	0.09	0.24	0.26
18:4	*	*	0.017	*	0.0074	*	*	*	*	*	*
19:0	*	*	0.04	*	*	*	*	*	*	*	*
19:1	*	*	0.17	*	*	*	*	*	*	*	*
19:2	*	*	0.014	*	*	*	*	*	*	*	*
20:0	*	0.89	0.044	0.19	0.11	0.24	0.13	*	0.03	*	*
20:1	*	*	0.48	0.14	*	0.08	*	*	0.28	*	*
20:2	*	*	0.22	*	0.16	0.40	*	*	0.37	*	*
20:3	*	0.6	0.23	0.01	0.45	3.25	*	*	3.4	4.09	3.28
20:4	*	0.36	0.68	*	1.35	10.97	*	*	10.61	8.98	11.4
20:5	*	0.66	0.107	0.34	0.20	0.84	*	*	1.65	1.03	0.56
22:0	*	*	0.015	0.48	0.074	0.64	0.23	*	*	*	*
22:1	*	*	0.042	*	0.013	*	*	*	*	*	*
22:2	*	*	0.008	*	0.0051	*	*	*	*	*	*
22:3	*	*	0.01	*	0.0018	*	*	*	*	*	*
22:4	*	1.74	0.12	*	0.17	0.41	*	*	0.33	*	*
22:5	*	*	0.22	0.41	0.18	1.18	*	*	1.45	1.25	*
22:6	*	*	0.50	1.49	0.46	3.37	*	*	5.41	4.03	2.76
**23:0**	*	*	*	*	**0.015**	*	**0.10**	*	*	*	*
24:0	*	*	0.02	*	0.12	0.46	0.22	*	*	*	*
24:1	*	*	0.045	*	0.032	0.78	*	*	*	*	*
Total MUFA	*	*	47.41	25.91	*	*	*	11.61	13.25	*	10
Total PUFA	*	*	13.70	*	*	*	*	*	*	*	42.7
n6-PUFA	*	7.77	11.3	*	*	*	*	38.24	38.06	*	*
n3-PUFA	*	1.01	2.24	2.62	*	*	*	7.81	*	*	*
Trans FA	*	*	*	*	*	*	*	0.1	*	*	*
Total OCFA	*	*	0.22	0.45	1.34	*	*	0.6	0.49	*	*
Total SFA	*	*	*	31.85	*	*	*	39.95	40.19	*	40.5
UNITS	% of total free fatty acids					% total phospholipid fatty acid

**Table:** 

(B)	CE					GL (TAG’s)				
Saturated Fatty Acid	*n* = 2657, [3]	*n* = 29, [31]	*n* = 3570, [32]	*n* = *, [25]	*n* = 25, [30]	*n* = 25, [30]	*n* = 7, [33]	*n* = 22, [35]	*n* = 8, [34]	*n* = 19, [36]
12:0	*	0	*	*	*	*	*	*	0.17	*
12:1	*	*	*	*	*	*	*	*	*	*
13:0	*	0	*	*	*	*	*	*	*	*
14:0	*	0.43	*	2.17	1.03	1.62	1.9	1.07	2.03	4.27
14:1	*	0.04	*	0.81	*	*	*	*	*	*
**15:0**	*	**0**	*	**0.81**	*	*	*	*	*	**0.67**
15:1	*	*	*	0.81	*	*	*	*	*	*
16:0	9.95	12.41	10.04	5.15	10.71	21.73	26.7	24.37	25.08	30.59
16:1	2.51	4.06	*	3.01	3.16	4.51	3.2	3.42	*	8.38
16:2	*	*	*	0.84	*	*	*	*	*	*
**17:0**	*	**0.04**	*	**0.87**	*	*	**0.8**	*	*	*
17:1	*	0.2	*	0.84	*	*	*	*	*	*
17:2	*	*	*	*	*	*	*	*	*	*
18:0	0.9	0.96	*	1.60	2.39	3.29	5.9	2.52	2.52	3.15
18:1	16	22.78	15.99	14.46	18.33	42.66	43.9	44.83	35.73	34.71
18:2	54.3	47.81	54.16	49.36	53.59	20.03	14.4	20.36	23.3	10.96
18:3	1.43	0.47	*	3.99	1.52	2.13	*	0.48	1.74	1.13
18:4	*	*	*	*	*	*	*	0	*	*
19:0	*	0	*	*	*	*	*	*	*	*
19:1	*	*	*	*	*	*	*	*	*	*
19:2	*	*	*	*	*	*	*	*	*	*
20:0	*	0.5	*	0.87	*	*	0.2	0.16	*	*
20:1	*	*	*	0.81	0.06	0.33	*	0.35	*	*
20:2	*	*	*	0.92	0.15	0.36	*	0	*	*
20:3	0.75	*	*	0.87	0.61	0.31	0.2	0	*	*
20:4	8.16	*	*	6.43	6.93	1.46	0.7	1.04	1.45	0.75
20:5	0.54	*	0.53	*	0.68	0.23	*	0	0.17	0.27
22:0	*	*	*	0.62	*	*	0.7	*	*	*
22:1	*	*	*	0.27	*	*	*	0	*	*
22:2	*	*	*	0.27	*	*	*	*	*	*
22:3	*	*	*	*	*	*	*	*	*	*
22:4	*	*	*	*	0.01	0.18	*	*	*	*
22:5	*	*	*	*	0.21	0.53	*	0.21	0.32	0.28
22:6	0.43	*	0.44	0.87	0.66	0.56	0.5	0.67	0.68	0.7
**23:0**	*	*	*	*	*	*	*	*	*	*
24:0	*	*	*	*	*	*	*	*	*	*
24:1	*	*	*	*	*	*	*	0.11	*	*
Total MUFA	18.6	*	18.59	*	*	*	*	48.71	40.99	*
Total PUFA	65.8	*	65.67	*	*	*	15.6	22.87	28.66	*
n6-PUFA	*	*	*	*	*	*	*	21.4	26.37	*
n3-PUFA	*	*	*	*	*	*	*	1.47	2.19	*
Trans FA	*	*	*	*	*	*	*	*	*	*
Total OCFA	*	*	*	3.34	*	*	*	*	*	*
Total SFA	11.6	*	11.69	*	*	*	37.5	28.12	30.27	*
UNITS	% of total cholesterol ester fatty acid					% of total glycerolipid fatty acid				

Note: * denotes data that was not indicated in the literature referenced.

Therefore, it seemed logical to use OCS-FAs as low cost internal standards in quantitative analysis, with C15:0 and C17:0 fatty acids being the most widely employed in this context. Many assumed that the concentration of OCS-FAs did not vary in different diseases and these lipid species were commonly used for standards in analyses [37,38]. The natural plasma variation of C17:0 could account for a 0.2%–3% variation in the Q-Int.Std response and therefore affecting the observed instrument abundance of the analyte (see Table 2). Furthermore the use of these two OCS-FAs as quantitative internal standards does not allow them to be incorporated into any statistical analysis and therefore no correlations can be deduced. This is the main limiting factor to the amount of understand there is around the physiology of OCS-FAs.

**Table 2:** The estimated error associated with the use of endogenous compounds containing. C15:0 and C17:0 as internal standards. For each study the added amount of internal standard and the possible sample endogenous concentration are shown, allowing the calculation of the possible error of the measured abundance of each of the fatty acid internal standards [25,39,40].

	Fatty Acid Internal Standard	Internal Standard and Sample Preparation	Int.Std Concentration in Sample	Sample Concentration of Fatty Acid	Sample Compound Error	References
1	15:0	50 µL of plasma was mixed with 25 µL of internal standard solution containing 3.01 µg of C15:0 in methanol, 1 mL of DMP, and 20 µL of concentrated aqueous HCL. Capped and kept at room temperature for 15 min. Pyridine, 10 µL, was added, then concentrated to 100 µL. Diluted with 0.5 mL of water. Aqueous mixture extracted with 0.5 mL of isooctane. After centrifugation, isooctane layer was transferred to a 1-mL serum, and evaporated to dryness. Then capped. Isooctane (30–50 µL) was added through the cap. An aliquot (0.5 µL) was injected into the gas chromatograph instrument.	60.2 µg/mL	≈0.158 µg/mL	0.26%	[37]
2	15:0	Blood is collected into a heparinized tube and centrifuged immediately at 4 °C in a refrigerated centrifuge. The plasma is removed and stored at −15 °C. Internal standard of 150 nMol C15:0 is added to 1.0 mL plasma. The FFA are extracted into 20 mL of extraction solvent by shaking for 30 s. The plasma and extraction solvent is left at room temperature for 15 min then mixed for 10 s. After centrifugation, the organic phase removed and evaporated to dryness. The dry residue contains the FFA and is stable for at least 24 h at 4 °C. The residue is dissolved in 100 µL *n*-heptane and 2 µL injected into the gas chromatograph instrument.	36.35 µg/mL	≈0.158 µg/mL	0.43%	[21]
3	17:0	Aliquots of 250 µL plasma were also placed into extraction tubes. A quantity of 50 µL of the C17:0 internal standard solution was spiked to each concentration standard and each plasma sample. The standards and plasma samples were extracted with freshly prepared Dole solution. The extracts were taken to dryness and were analyzed on the LC-MS. One tenth of the volume of each concentration standard and each plasma sample were re-suspended in 400 µL of buffer A (80% acetonitrile, 0.5 mM ammonium acetate) prior to injecting 10 µL onto the LC-MS.	12 µg/mL	≈0.323 µg/mL	2.69%	[38]

### 2.2. Biomarkers for Dietary Food Intake

With the realization that OCS-FAs are in fact a biologically relevant component of blood plasma [41] there came further insights into their origin, either through consumption or through endogenous biosynthetic or metabolic pathways. This new direction of research interest led into the field of dietary analysis and the aim to identify lipidome variations [42] in relation to dietary intake [43,44,45]. OCS-FAs have attracted attention with research into the possible application of C15:0 in blood as a marker for intake of milk fat [26] and subsequent relations between intake of milk fat with metabolic risk factors, the results in the first published study that focused on this showed that the proportions of C15:0 in cholesterol esters are associated with the total amount of fat from milk products (r = 0.46, *p* < 0.0001), based on 62 men [46]. 

The reason that OCS-FAs are thought to mainly originate from dairy fat [26,47] is based on the observation that these fatty acids are produced in relatively high levels by rumen microbial fermentation and microbial *de-novo* lipogenesis [4] which then transfers into the host animal. Likewise with mammals, microbial *de-novo* lipogenesis is the act of repeated condensation of malonyl CoA with acetyl CoA as a starting compound [48]; the sequential condensation reactions predominantly produce hexadecanoic acid (C16:0) and to a lesser extent [4] octadecanoic acid (C18:0) (both even chain length fatty acids) depending on the microbial species population ratio (Protozoa: Bacteria) [6,49]. Alternatively, in certain microbes C17:0 can be produced where propionic acid, a volatile fatty acid, is trapped by the rumen bacteria/protozoa and can be used in *de novo* lipogenesis [50,51,52]. OCS-FAs can also be produced by the rumen microbial population via a different pathway which utilizes the removal of the α-carbon [18,53], through the conversion of C16:0 or C18:0 (end products of *de novo* lipogenesis) to a hydroxyl fatty acid followed by decarboxylation to produce either C15:0 or C17:0, respectively. This results in up to a 40% (depending on the bacterial species [4]) OCS-FAs content within the cells, with the remainder being predominantly C16:0 and C18:0.

**Figure 1:**
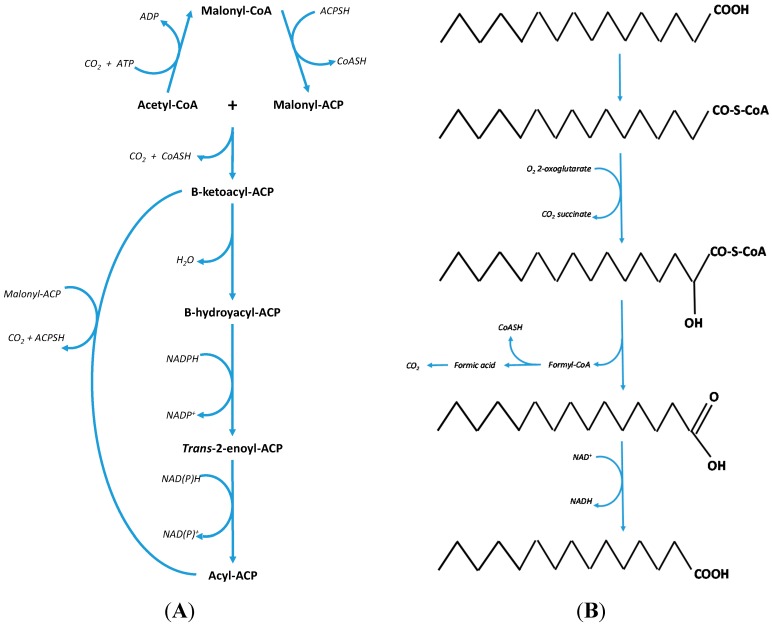
(**A**) On the left—the bacteria bio-synthesis pathway for the production of the fatty acids, C16:0 and C18:0 through the repeated condensation of malonyl CoA with acetyl CoA [19]. (**B**) On the right—the fundamental processes of α-oxidation where the removal of one carbon produces an odd chain fatty acid [18].

These OCS-FAs are then taken up by the animal from the rumen and utilized by the mammary gland for the production of milk fat. The resulting level of OCS-FAs in milk fat is only between 1.5%–2.5% [54,55]. The ratio of C15:0 to C17:0 is approximately 2:1 [56,57] within ruminant milk fat in part due to the more abundant production of C16:0 over C18:0 during *de novo* lipogenesis. In addition to this the apparent oxidation of the individual fatty acids decreases with an increase in carbon chain length [58] resulting in this approximate 2:1 ratio. The process of α-oxidation is summarized in Figure 1B. 

Dairy fat intake has been positively correlated with an increase in plasma saturated fatty acids [59] and on this basis one might expect that there would be a negative association between dairy fat and cardiovascular health however recent evidence contradicts this assumption. In a number of studies, saturated fatty acids have been proven to be detrimental to health, in part associated with their effect on cholesterol metabolism as well as direct factors associated with disease [7]. On the contrary, C15:0 and C17:0 have been shown to have a positive association with health which relate to several disease etiologies [12,60]. Holman and colleagues [61] described that both C15:0 and C17:0 have an association with reduced risk for developing multiple sclerosis with it being suggested that the fatty acids are thought to increase the fluidity of membranes [62] to a similar degree as polyunsaturated fatty acids. The authors hypothesized that the OCS-FAs are important to meet the homeostatic range compatible with the necessary requirements of membrane functionality [61]. Currently there is a large amount of research going into the role of polyunsaturated fatty acids (PUFAs) in Alzheimer’s disease with [63,64,65] where they have been found to have two key roles; firstly neurotransmission and prostaglandin formation [66] and secondly for the improvement of membrane fluidity [67]. In a recently published manuscript by Fonteh and colleagues [68] it was shown that tissue levels of OCS-FAs were lower in Alzheimer’s disease when compared to a control group. With the realisation that OCS-FAs increase membrane fluidity more than PUFAs then the application of OCS-FAs as a form of treatment for Alzheimer’s disease could be a possibility. Interestingly, OCS-FAs are compartmentalized within tissue [68,69] and therefore can be distinguished from other fatty acids adding to their interest in research. In addition to this, OCS-FAs may have an anti-carcinogenic influence on cancer cells which further adds to the reason for their research interest within public health nutrition [4].

### 2.3. Predictor Biomarkers for Coronary Heart Disease (CHD) and Type II Diabetes (T2D)

In recent years research has been carried out in two key studies: The European Prospective Investigation into Cancer and Nutrition (EPIC) and The Norfolk Prospective Study [12]. The plasma samples of 1595 CHD cases and 2246 controls were used to extract plasma phospholipid fatty acids. The lipid extracts were measured by gas chromatography coupled to electron impact mass spectrometry and the concentrations were determined by peak comparison with an internal standard (di-palmitoyl-D31-phosphatidylcholine). The incidence of CHD was ascertained by the participant’s admission into hospital with a CHD diagnosis or death from CHD according to ICD9 410-414/ICD10 I22–I25. The results from this study clearly revealed saturated plasma phospholipid fatty acid, C14:0, C16:0, C18:0, concentrations were significantly associated with an increased risk of CHD. However, OCS-FAs concentrations of C15:0 and C17:0 showed a significant inverse association with CHD incidence. 

More recent work based on the EPIC and INTERACT studies [27] examined the association between the incidence of T2D and the initial plasma phospholipid fatty acids, specifically C14:0, C15:0, C16:0, C17:0 and C18:0 which were measured in 12,403 T2D cases and 16,154 controls. This showed that saturated even numbered fatty acids from plasma phospholipids have a strong positive association with T2D incidence whereas plasma phospholipid OCS-FAs showed a strong inverse association with disease risk. Table 3 shows the testing characteristics of ten unique studies focusing on C15:0 and C17:0 with regards to disease risk. 

**Table 3:** Data collated from the literature regarding C15:0 and C17:0 with their association to disease risk, biomarker identification or treatment pathway. This is not a meta-analysis but an illustration that odd chain fatty acids have been associated in several diseases including metabolic and psychological pathologies.

	Study Disease	Number of Participants	Country	Outcome	Study
1	Disorders of propionate, methylmalonate and biotin metabolism	24 diseased 12 control	Netherlands	OCS-FAs—disease treatment marker	[13]
2	Atherosclerosis	2837 cohort	USA	OCS-FAs—inverse relationship with disease development	[70]
3	Type II diabetes	346 diseased 3391 control	Australia	OCS-FAs—inverse relationship with disease development	[71]
4	Coronary Heart Disease	1595 diseased 2246 control	UK	OCS-FAs—inverse relationship with disease development	[12]
5	Prediabetes and Type II Diabetes	181 diseased 170 control	Australia	OCS-FAs—inverse relationship with disease development	[60]
6	Biotin Deficiency	3 diseased	USA	OCS-FAs—increased in diseased cases	[72]
7	Peroxisomal Disorders	86 diseased 84 control	USA	OCS-FAs—increased in diseased cases	[73]
8	Insulin sensitivity	86 diseased	Australia	OCS-FAs—inverse relationship with disease development	[20]
9	Cardiomyopathy and rhabdomyolysis in long-chain fat oxidation disorders	107 disease 50 control	USA	OCS-FAs—improve disease prognosis	[74]
10	Anorexia Nervosa	8 diseased 19 control	USA	OCS-FAs—improves cell membrane fluidity	[62]

This table displays the country, the number of participants, the studied disease and the outcome of that research. The aim of the table is not to provide a comprehensive meta-analysis but to show that many studies have claimed that there is an association between OCS-FAs and different pathologies. It will remain to be seen if a meta-analysis will proof that this association is true.

### 2.4. Evidence for Alternative Metabolic Pathways

**Figure 2:**
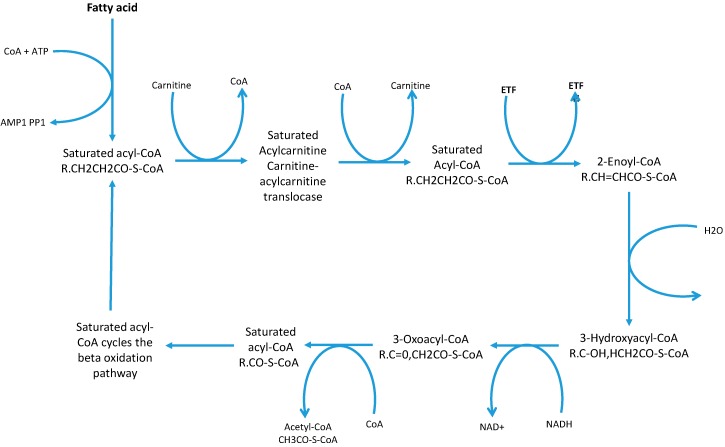
Cyclic β-oxidation process and the production of the acetyl CoA molecules [76]. This diagram shows the substrates and products of each reaction in the β-oxidation pathway.

It is generally assumed that OCS-FAs are totally derived from dietary consumption of milk and other dairy products and originate from bio-synthesis in rumen microbiome. This assumption is eroding slowly [24] due to further understanding of alternative metabolic pathways which started with research into certain genetic diseases, such as Refsum disease and Zellweger Syndrome [18] were there was an accumulation of phytanic acid (3,7,10,14-tetramethylhexadecanoic acid) a β-branched-chain fatty acid. β-Branched chain fatty acids cannot undergo β-oxidation [75] and therefore need an alternative metabolic route to avoid compound accumulation. Additionally, this is seen in some genetic mutations where these alternative pathways are impaired and result in an accumulation of β-branched chain fatty acids. Typically fatty acids undergo β-oxidation; defined as the degradation of the fatty acid chain by units of acetyl CoA molecules producing NADH and FADH2 which are processed to produce ATP in the electron transport chain [76] (see Figure 2). 

**Figure 3:**
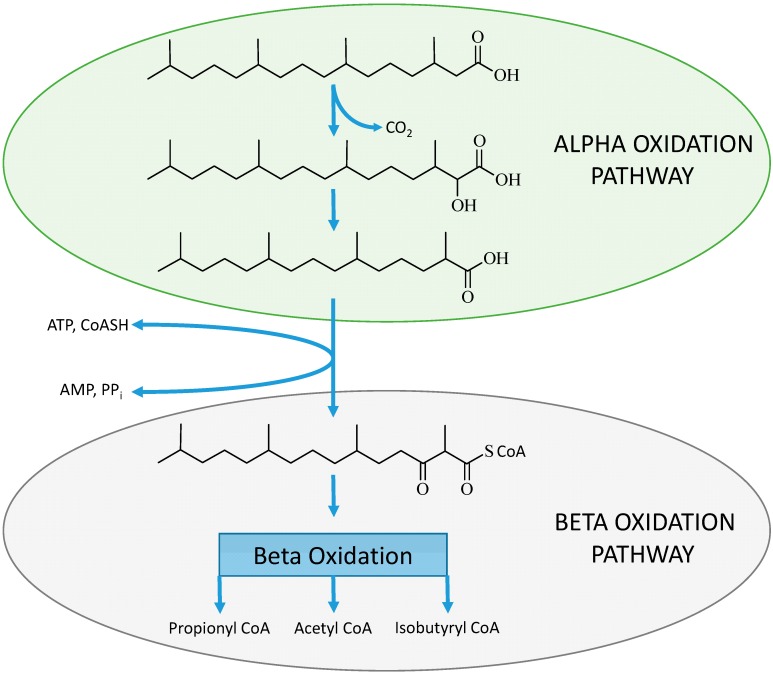
The α-oxidation process on the β-branched chain fatty acid (phytanic acid) to produce an α-branched chain fatty acid (pristanic acid) which then can be activated and enter the β-oxidation pathway [77].

In phytanic acid an acetyl CoA molecule cannot be removed due to the presence of a methyl group on the β-carbon position and therefore these β-branched chain fatty acids must undergo alternative oxidation pathways [77] to avoid physiological accumulation. The work of Wanders *et al.* described the process of α-oxidation of these molecules as an alternative pathway for the oxidation of β-branched chain fatty acids [78]. The α-oxidation process (see Figure 1B) involves the activation of the fatty acid then hydroxylation of the α-carbon in relation to the terminal carboxylic acid, which requires iron and α-keto-glutarate as co-factors. This step is then followed by the removal of the terminal carboxyl group involving thymine pyrophosphate and magnesium ions [18]. The conversion of the α-hydroxyl group to a terminal carboxyl group results in an α-branched chain fatty acid that can directly undergo β-oxidation (see Figure 3).

This process has been proven to occur in humans but was thought to act only on β-branched chain fatty acids and not to occur on straight chain fatty acids due to the obscurity of the reaction and the localization of the reaction to the peroxisome cell organelle. Contemporarily, there has been emerging evidence that α-oxidation may operate on straight chain fatty acids to produce OCS-FAs [79,80,81,82,83,84,85], this evidence is summarized in the following sections. 

#### 2.4.1. Inconsistent Ratios of C15:0 to C17:0 when Comparing Lipid Consumption with Measured Plasma Levels

**Figure 4:**
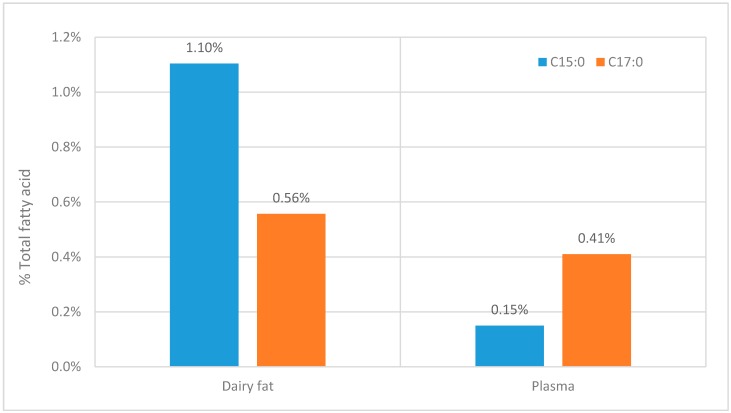
The comparison between the C15:0 and C17:0 fatty acids within dairy products [4] and the comparative concentration within in plasma [59].

Although Table 1 shows that many studies lack useful information on OCS-FAs it is clear that there is a consistent difference between the C15:0 and C17:0 fatty acids. The ratio C15:0 to C17:0 is approximately 1:2 and this seems to be across all the lipid classes, although for some classes there is less information given (see Table 1). This ratio contradicts the ratio of these fatty acid in the diet (see Figure 4). When it would be expected that when OCS-FAs solely originates from dairy fat, the ratio between C15:0 and C17:0 should be at least similar to the ratio found in dairy fat.

Within the wider literature there is a similar trend, where C17:0 dominates as the most abundant OCS-FAs followed by C15:0 when analyzed within other human tissue tissues and biofluid, serum (C15:0 of 0.22% *, C17:0 of 0.37% *) [47], adipose tissue (C15:0 of 0.32% *, C17:0 of 0.34% *) [47], Erythrocyte (C15:0 of 0.28% *, C17:0 of 0.45% *) and human hindmilk [86] (C15:0 of 0.46% *, C17:0 of 0.57% *). NB.* = % of total fatty acid.

#### 2.4.2. Bio-Synthesized Odd Chain Fatty Acids in Adipocyte Differentiation

In the study by Roberts *et al.* [87] on the differentiation of adipocytes a significant increase of OCS-FAs occurred. This provided evidence showing that straight chain fatty acids can be metabolized through an α-oxidation pathway. The differentiating adipocytes were able to convert C16:0 labeled fatty acid with a stable isotope to C15:0 still exhibiting the stable isotope label. This only occurred in the cells and not in the medium, which showed that OCS-FAs were endogenously metabolizing C16:0 into C15:0 [87]. These results show that α-oxidation occurs on straight chain fatty acids producing a single-carbon atom abatement and therefore an OCS-FAs.

## 3. Conclusions

From the literature it can be concluded that there is an association between plasma OCS-FAs and dietary intake of dairy fat, and this can contribute to the discussion that the consumption of OCS-FAs containing foods, such as dairy fats, could reduce the risk of developing metabolic diseases. On the other hand, there is at the moment no decisive evidence for a direct relation between both C15:0 and C17:0 plasma concentrations reflecting just dietary consumptions. This suggests that there could be other factors that need to be taken into account before a public health message can be formulated.

Different large scale epidemiological studies have now shown that the plasma OCS-FAs levels are associated with reduced disease risks for CHD [12,74,88,89] and T2D [27,90]. These studies contradict the original ideas around OCS-FAs being insignificant in comparison to even chain fatty acids. This also raises new research questions on cooperative nutrient consumption (does propionyl CoA production through branched chain amino acid metabolism increases De novo OCS-FA synthesis [48]), endogenous metabolic reactions (does endogenous α-oxidation play a role in lipid metabolism) and genetic ascendancy (propionic acidemia [91]) on the plasma phospholipid OCS-FAs with regards to disease pathology, and these urgently requires further research.

With regards to OCS-FAs there is need for caution because there has been very little research into any possible negative effects of high consumption but two aspects have arisen, for example behavioural maturation and hepatic oxidation inhibition. Research by Gozzo *et al.* has shown that OCS-FAs are capable of passing through the placental barrier and into milk of lactating mammals [92], this leads to the possibility that these fatty acids are also capable of crossing the cerebral endothelium (blood-brain barrier) and act on gestational and early postnatal brain development. Since brain microsomes are already known to perform α-oxidation reactions [16] then any variation in the flux of OCS-FAs may disrupt this process. Hepatic short chain fatty acid oxidation inhibition is a lesser concern due to the endogenous synthesis of carnitine which acts on the end product of OCS-FAs metabolism, propionyl CoA, reducing it by 50%, but as the subject increases in age the biosynthesis of carnitine reduces and propionyl CoA associated hepatic inhibition of ketogenesis may become an issue [93].

To summarize, it is clear that C15:0 and C17:0 can be utilized as rough markers for dairy fat intake with regards to dietary analysis but the main area of interest is with the identification of an alternate pathways, such as α-oxidation since this incites an additional area of research within metabolic pathology.

## References

[1] Miettinen T.A., Railo M., Lepäntalo M., Gylling H. (2005). Plant sterols in serum and in atherosclerotic plaques of patients undergoing carotid endarterectomy. J. Am. Coll. Cardiol..

[2] Manninen V., Tenkanen L., Koskinen P., Huttunen J.K., Mänttäri M., Heinonen O.P., Frick M.H. (1992). Joint effects of serum triglyceride and LDL cholesterol and HDL cholesterol concentrations on coronary heart disease risk in the helsinki heart study. Implications for treatment. Circulation.

[3] Wang L., Folsom A.R., Zheng Z.J., Pankow J.S., Eckfeldt J.H. (2003). ARIC study investigators. Plasma fatty acid composition and incidence of diabetes in middle-aged adults: The atherosclerosis risk in communities (ARIC) study. Am. J. Clin. Nutr..

[4] Vlaeminck B., Fievez V., Cabrita A.R.J., Fonseca A.J.M., Dewhurst R.J. (2006). Factors affecting odd- and branched-chain fatty acids in milk: A review. Anim. Feed Sci. Technol..

[5] Reitz C., Tang M., Luchsinger J., Mayeux R. (2004). Relation of plasma lipids to alzheimer disease and vascular dementia. Arch. Neurol..

[6] LIPID Maps. http://www.lipidmaps.org/.

[7] Ulbricht T.L.V., Southgate D.A.T. (1991). Coronary heart disease: Seven dietary factors. Lancet.

[8] Simopoulos A.P. (1991). Omega-3 fatty acids in health and disease and in growth and development. Am. J. Clin. Nutr..

[9] Izai K., Uchida Y., Orii T., Yamamoto S., Hashimoto T. (1992). Novel fatty acid beta-oxidation enzymes in rat liver mitochondria. I. Purification and properties of very-long-chain acyl-coenzyme a dehydrogenase. J. Biol. Chem..

[10] Poulos A., Sharp P., Fellenberg A.J., Danks D.M. (1985). Cerebro-hepato-renal (zellweger) syndrome, adrenoleukodystrophy, and refsum’s disease: plasma changes and skin fibroblast phytanic acid oxidase. Hum. Genet..

[11] Hodson L., Skeaff C.M., Fielding B.A. (2008). Fatty acid composition of adipose tissue and blood in humans and its use as a biomarker of dietary intake. Prog. Lipid Res..

[12] Khaw K.T., Friesen M.D., Riboli E., Luben R., Wareham N. (2012). Plasma phospholipid fatty acid concentration and incident coronary heart disease in men and women: The EPIC-norfolk prospective study. PLoS Med..

[13] Çoker M., de Klerk J.B.C., Poll-The B.T., Huijmans J.G.M., Duran M. (1996). Plasma total odd-chain fatty acids in the monitoring of disorders of propionate, methylmalonate and biotin metabolism. J. Inherit. Metab. Dis..

[14] Phillips G.B., Dodge J.T. (1967). Composition of phospholipids and of phospholipid fatty acids of human plasma. J. Lipid Res..

[15] Horning M.G., Martin D.B., Karmen A., Vagelos P.R. (1961). Fatty acid synthesis in adipose tissue II. Enzymatic synthesis of branched chain and odd-numbered fatty acids. J. Biol. Chem..

[16] Mead J.F., Gabriel M. (1963). Levis. A 1 Carbon degradation of the long chain fatty acids of brain sphingolipids. J. Biol. Chem..

[17] Vanitallie T.B., Khachadurian A.K. (1969). Rats enriched with odd-carbon fatty acids: Maintenance of liver glycogen during starvation. Science.

[18] Jansen G.A., Ronald J.A. (2006). Wanders. Alpha-oxidation. Biochim. Biophys. Acta (BBA) Mol. Cell Res..

[19] Ferrannini E., Barrett E.J., Bevilacqua S., DeFronzo R.A. (1983). Effect of fatty acids on glucose production and utilization in man. J. Clin. Investig..

[20] Nestel P.J., Straznicky N., Mellett N.A., Wong G., De Souza D.P., Tull D.L., Barlow C.K., Grima M.T., Meikle P.J. (2014). Specific plasma lipid classes and phospholipid fatty acids indicative of dairy food consumption associate with insulin sensitivity. Am. J. Clin. Nutr..

[21] Sampson D., Hensley W.J. (1975). A rapid gas chromatographic method for the quantitation of underivatised individual free fatty acids in plasma. Clin. Chim. Acta.

[22] Novgorodtseva T.P., Karaman Y.K., Zhukova N.V., Lobanova E.G., Antonyuk M.V., Kantur T.A. (2011). Composition of fatty acids in plasma and erythrocytes and eicosanoids level in patients with metabolic syndrome. Lipids Health Dis..

[23] Hellmuth C., Demmelmair H., Schmitt I., Peissner W., Blüher M., Koletzko B. (2013). Association between plasma nonesterified fatty acids species and adipose tissue fatty acid composition. PLoS One.

[24] Baylin A., Kim M.K., Donovan-Palmer A., Siles X., Dougherty L., Tocco P., Campos H. (2005). Fasting whole blood as a biomarker of essential fatty acid intake in epidemiologic studies: comparison with adipose tissue and plasma. Am. J. Epidemiol..

[25] Quehenberger O., Armando A.M., Brown A.H., Milne S.B., Myers D.S., Merrill A.H., Bandyopadhyay S., Jones K.N., Kelly S., Shaner R.L. (2010). Lipidomics reveals a remarkable diversity of lipids in human plasma. J. Lipid Res..

[26] Matthan N.R., Ooi E.M., van Horn L., Neuhouser M.L., Woodman R., Lichtenstein A.H. (2014). Plasma phospholipid fatty acid biomarkers of dietary fat quality and endogenous metabolism predict coronary heart disease risk: A nested case-control study within the women’s health initiative observational study. J. Am. Heart Assoc..

[27] Forouhi N.G., Koulman A., Sharp S.J., Imamura F., Kröger J., Schulze M.B., Crowe F.L., Huerta J.M., Guevara M., Beulens J.W. (2014). Differences in the prospective association between individual plasma phospholipid saturated fatty acids and incident type 2 diabetes: The EPIC-InterAct case-cohort study. Lancet Diabetes Endocrinol..

[28] Saadatian-Elahi M., Slimani N., Chajès V., Jenab M., Goudable J., Biessy C., Ferrari P., Byrnes G., Autier P., Peeters P.H. (2009). Plasma phospholipid fatty acid profiles and their association with food intakes: Results from a cross-sectional study within the European prospective investigation into cancer and nutrition. Am. J. Clin. Nutr..

[29] Crowe F.L., Allen N.E., Appleby P.N., Overvad K., Aardestrup I.V., Johnsen N.F., Tjønneland A., Linseisen J., Kaaks R., Boeing H. (2008). Fatty acid composition of plasma phospholipids and risk of prostate cancer in a case-control analysis nested within the European prospective investigation into cancer and nutrition. Am. J. Clin. Nutr..

[30] Zák A., Vecka M. (2005). Composition of plasma fatty acids and non-cholesterol sterols in anorexia nervosa. Physiol. Res. Acad. Sci. Bohemoslov..

[31] Dyerberg J., Bang H.O., Hjorne N. (1975). Fatty acid composition of the plasma lipids in Greenland Eskimos. Am. J. Clin. Nutr..

[32] Ma J., Folsom A.R., Shahar E., Eckfeldt J.H. (1995). Plasma fatty acid composition as an indicator of habitual dietary fat intake in middle-aged adults. The atherosclerosis risk in communities (ARIC) study investigators. Am. J. Clin. Nutr..

[33] Caramia G., Cocchi M. (2007). Fatty acids composition of plasma phospholipids and triglycerides in children with cystic fibrosis. The effect of dietary supplementation with an olive and soybean oils mixture. Pediatr. Med. E Chir. Med. Surg. Pediatr..

[34] Raatz S.K., Bibus D., Thomas W., Kris-Etherton P. (2001). Total fat intake modifies plasma fatty acid composition in humans. J. Nutr..

[35] Ruíz-Gutiérrez V., Prada J.L., Pérez-Jiménez F. (1993). Determination of fatty acid and triacylglycerol composition of human very-low-density lipoproteins. J. Chromatogr. B Biomed. Sci. Appl..

[36] Skeaff C.M., Hodson L., McKenzie J.E. (2006). Dietary-induced changes in fatty acid composition of human plasma, platelet, and erythrocyte lipids follow a similar time course. J. Nutr..

[37] Tserng K.Y., Kliegman R.M., Miettinen E.L., Kalhan S.C. (1981). A rapid, simple, and sensitive procedure for the determination of free fatty acids in plasma using glass capillary column gas-liquid chromatography. J. Lipid Res..

[38] Persson X.M., Blachnio-Zabielska A.U., Jensen M.D. (2010). Rapid measurement of plasma free fatty acid concentration and isotopic enrichment using LC/MS. J. Lipid Res..

[39] Kagan M.L., West A.L., Zante C., Calder P.C. (2013). Acute appearance of fatty acids in human plasma—A comparative study between polar-lipid rich oil from the microalgae nannochloropsis oculata and krill oil in healthy young males. Lipids Health Dis..

[40] Moser A.B., Kreiter N., Bezman L., Lu S., Raymond G.V., Naidu S., Moser H.W. (1999). Plasma very long chain fatty acids in 3000 peroxisome disease patients and 29,000 controls. Ann. Neurol..

[41] Astrup A. (2014). A changing view on saturated fatty acids and dairy: From enemy to friend. Am. J. Clin. Nutr..

[42] Seppänen-Laakso T., Oresic M. (2009). How to study lipidomes. J. Mol. Endocrinol..

[43] Emmanuel B. (1978). The relative contribution of propionate, and long-chain even-numbered fatty acids to the production of long-chain odd-numbered fatty acids in rumen bacteria. Biochim. Biophys. Acta (BBA) Lipids Lipid Metab..

[44] Hughes R. (2003). Definitions for public health nutrition: A developing consensus. Public Health Nutr..

[45] Jeremiah S. (1982). Diet and Coronary Heart Disease. Proceedings of “Current Topics in Biostatistics and Epidemiology”. A Memorial Symposium in Honor of Jerome Cornfield. Biometrics.

[46] Smedman A.E., Gustafsson I.B., Berglund L.G., Vessby B.O. (1999). Pentadecanoic acid in serum as a marker for intake of milk fat: Relations between intake of milk fat and metabolic risk factors. Am. J. Clin. Nutr..

[47] Brevik A., Veierød M.B., Drevon C.A., Andersen L.F. (2005). Evaluation of the odd fatty acids 15:0 and 17:0 in serum and adipose tissue as markers of intake of milk and dairy fat. Eur. J. Clin. Nutr..

[48] Laliotis G.P., Bizelis I., Rogdakis E. (2010). Comparative approach of the de novo fatty acid synthesis (lipogenesis) between ruminant and non-ruminant mammalian species: From biochemical level to the main regulatory lipogenic genes. Curr. Genomics.

[49] Or-Rashid M.M., Odongo N.E., McBride B.W. (2007). Fatty acid composition of ruminal bacteria and protozoa, with emphasis on conjugated linoleic acid, vaccenic acid, and odd-chain and branched-chain fatty acids. J. Anim. Sci..

[50] Dijkstra J., van Zijderveld S.M., Apajalahti J.A., Bannink A., Gerrits W.J.J., Newbold J.R., Perdok H.B., Berends H. (2011). Relationships between methane production and milk fatty acid profiles in dairy cattle. Anim. Feed Sci. Technol..

[51] French E.A., Bertics S.J., Armentano L.E. (2012). Rumen and milk odd- and branched-chain fatty acid proportions are minimally influenced by ruminal volatile fatty acid infusions. J. Dairy Sci..

[52] Heck J.M., van Valenberg H.J., Bovenhuis H., Dijkstra J., van Hooijdonk T. (2012). Characterization of milk fatty acids based on genetic and herd parameters. J. Dairy Res..

[53] Berthelot V., Bas P., Pottier E., Normand J. (2012). The effect of maternal linseed supplementation and/or lamb linseed supplementation on muscle and subcutaneous adipose tissue fatty acid composition of indoor lambs. Meat Sci..

[54] Dohme-Meier F., Bee G. (2012). Feeding unprotected cla methyl esters compared to sunflower seeds increased milk CLA level but inhibited milk fat synthesis in cows. Asian Australas J. Anim. Sci..

[55] Stefanov I., Baeten V., Abbas O., Vlaeminck B., De Baets B., Fievez V. (2013). Evaluation of FT-NIR and ATR-FTIR spectroscopy techniques for determination of minor odd- and branched-chain saturated and trans unsaturated milk fatty acids. J. Agric. Food Chem..

[56] Dewhurst R.J., Moorby J.M., Vlaeminck B., Fievez V. (2007). Apparent recovery of duodenal odd- and branched-chain fatty acids in milk of dairy cows. J. Dairy Sci..

[57] Fievez V., Colman E., Castro-Montoya J.M., Stefanov I., Vlaeminck B. (2012). Milk odd- and branched-chain fatty acids as biomarkers of rumen function—An update. Anim. Feed Sci. Technol..

[58] James P.D., Windhauser M.M., Champagne C.M., Bray G.A. (2000). Differential oxidation of individual dietary fatty acids in humans. Am. J. Clin. Nutr..

[59] Sun Q., Ma J., Campos H., Hu F.B. (2007). Plasma and erythrocyte biomarkers of dairy fat intake and risk of ischemic heart disease. Am. J. Clin. Nutr..

[60] Meikle P.J., Wong G., Barlow C.K., Weir J.M., Greeve M.A., MacIntosh G.L., Almasy L., Comuzzie A.G., Mahaney M.C., Kowalczyk A. (2013). Plasma lipid profiling shows similar associations with prediabetes and type 2 diabetes. PLoS One.

[61] Holman R.T., Johnson S.B., Kokmen E. (1989). Deficiencies of polyunsaturated fatty acids and replacement by nonessential fatty acids in plasma lipids in multiple sclerosis. Proc. Natl. Acad. Sci. USA.

[62] Holman R.T., Adams C.E., Nelson R.A., Grater S.J., Jaskiewicz J.A., Johnson S.B., Erdman J.W. (1995). Patients with anorexia nervosa demonstrate deficiencies of selected essential fatty acids, compensatory changes in nonessential fatty acids and decreased fluidity of plasma lipids. J. Nutr..

[63] Bazinet R.P., Layé S. (2014). Polyunsaturated fatty acids and their metabolites in brain function and disease. Nat. Rev. Neurosci..

[64] Tan Z.S., Harris W.S., Beiser A.S., Au R., Himali J.J., Debette S., Pikula A., DeCarli C., Wolf P.A., Vasan R.S. (2012). Red blood cell omega-3 fatty acid levels and markers of accelerated brain aging. Neurology.

[65] Torres M., Price S.L., Fiol-deRoque M.A., Marcilla-Etxenike A., Ahyayauch H., Barceló-Coblijn G., Terés S., Katsouri L., Ordinas M., López D.J. (2014). Membrane lipid modifications and therapeutic effects mediated by hydroxydocosahexaenoic acid on Alzheimer’s disease. Biochim. Biophys. Acta (BBA) Biomembr..

[66] Haag M. (2003). Essential fatty acids and the brain. Can. J. Psychiatry.

[67] Yang X., Sun G.Y., Eckert G.P., Lee J.C.-M. (2014). Cellular membrane fluidity in amyloid precursor protein processing. Mol. Neurobiol..

[68] Fonteh A.N., Cipolla M., Chiang J., Arakaki X., Harrington M.G. (2014). Human cerebrospinal fluid fatty acid levels differ between supernatant fluid and brain-derived nanoparticle fractions, and are altered in Alzheimer’s disease. PLoS One.

[69] Shibata R., Gotoh N., Kubo A., Kanda J., Nagai T., Mizobe H., Yoshinaga K., Kojima K., Watanabe H., Wada S. (2012). Comparison of catabolism rate of fatty acids to carbon dioxide in mice. Eur. J. Lipid Sci. Technol..

[70] De Oliveira Otto M.C., Nettleton J.A., Lemaitre R.N., Steffen L.M., Kromhout D., Rich S.S., Tsai M.Y., Jacobs D.R., Mozaffarian D. (2013). Biomarkers of dairy fatty acids and risk of cardiovascular disease in the multi-ethnic study of atherosclerosis. J. Am. Heart Assoc. Cardiovasc. Cerebrovasc. Dis..

[71] Hodge A.M., English D.R., O’Dea K., Sinclair A.J., Makrides M., Gibson R.A., Giles G.G. (2007). Plasma phospholipid and dietary fatty acids as predictors of type 2 diabetes: Interpreting the role of linoleic acid. Am. J. Clin. Nutr..

[72] Mock D.M., Johnson S.B., Holman R.T. (1988). Effects of biotin deficiency on serum fatty acid composition: evidence for abnormalities in humans. J. Nutr..

[73] Moser H.W., Moser A.B., Frayer K.K., Chen W., Schulman J.D., O’Neill B.P., Kishimoto Y. (1981). Adrenoleukodystrophy increased plasma content of saturated very long chain fatty acids. Neurology.

[74] Roe C.R., Sweetman L., Roe D.S., David F., Brunengraber H. (2002). Treatment of cardiomyopathy and rhabdomyolysis in long-chain fat oxidation disorders using an anaplerotic odd-chain triglyceride. J. Clin. Investig..

[75] Mannaerts G.P., Van Veldhoven P.P., Casteels M. (2000). Peroxisomal lipid degradation via beta- and alpha-oxidation in mammals. Cell Biochem. Biophys..

[76] Eaton S., Bartlett K., Pourfarzam M. (1996). Mammalian mitochondrial beta-oxidation. Biochem. J..

[77] Wierzbicki A.S., Lloyd M.D., Schofield C.J., Feher M.D., Gibberd F.B. (2002). Refsum’s disease: A peroxisomal disorder affecting phytanic acid α-oxidation. J. Neurochem..

[78] Wanders R.J., Jansen G.A., Lloyd M.D. (2003). Phytanic acid alpha-oxidation, new insights into an old problem: A review. Biochim. Biophys. Acta (BBA) Mol. Cell Biol. Lipids.

[79] Foulon V., Sniekers M., Huysmans E., Asselberghs S., Mahieu V., Mannaerts G.P., Veldhoven P.P.V., Casteels M. (2005). Breakdown of 2-hydroxylated straight chain fatty acids via peroxisomal 2-hydroxyphytanoyl-coa lyase a revised pathway for the α-oxidation of straight chain fatty acids. J. Biol. Chem..

[80] Guo L., Zhou D., Pryse K.M., Okunade A.L., Su X. (2010). Fatty acid 2-hydroxylase mediates diffusional mobility of raft-associated lipids, glut4 level, and lipogenesis in 3t3-l1 adipocytes. J. Biol. Chem..

[81] Kondo N., Ohno Y., Yamagata M., Obara T., Seki N., Kitamura T., Naganuma T., Kihara A. (2014). Identification of the phytosphingosine metabolic pathway leading to odd-numbered fatty acids. Nat. Commun..

[82] Nagy K., Brahmbhatt V.V., Berdeaux O., Bretillon L., Destaillats F., Acar N. (2012). Comparative study of serine-plasmalogens in human retina and optic nerve: Identification of atypical species with odd carbon chains. J. Lipid Res..

[83] Su X., Han X., Yang J., Mancuso D.J., Chen J., Bickel P.E., Gross R.W. (2004). Sequential ordered fatty acid α oxidation and δ9 desaturation are major determinants of lipid storage and utilization in differentiating adipocytes. Biochemistry.

[84] Veldhoven P.P.V. (2010). Biochemistry and genetics of inherited disorders of peroxisomal fatty acid metabolism. J. Lipid Res..

[85] Yuki D., Sugiura Y., Zaima N., Akatsu H., Hashizume Y., Yamamoto T., Fujiwara M., Sugiyama K., Setou M. (2011). Hydroxylated and non-hydroxylated sulfatide are distinctly distributed in the human cerebral cortex. Neuroscience.

[86] Valentine N.H., Richard S. (1994). Hindmilk Improves Weight Gain in Low-Birth-Weight Infants Fe: Journal of Pediatric Gastroenterology and Nutrition. J. Pediatr. Gastr. Nutr..

[87] Roberts L.D., Virtue S., Vidal-Puig A., Nicholls A.W., Griffin J.L. (2009). Metabolic phenotyping of a model of adipocyte differentiation. Physiol. Genomics.

[88] Labarthe F., Gélinas R., Rosiers C.D. (2008). Medium-chain fatty acids as metabolic therapy in cardiac disease. Cardiovasc. Drugs Ther..

[89] Jacobs S., Schiller K., Jansen E., Fritsche A., Weikert C., di Giuseppe R., Boeing H., Schulze M.B., Kröger J. (2014). Association between erythrocyte membrane fatty acids and biomarkers of dyslipidemia in the EPIC-Potsdam study. Eur. J. Clin. Nutr..

[90] Mozaffarian D. (2014). Saturated fatty acids and type 2 diabetes: More evidence to re-invent dietary guidelines. Lancet Diabetes Endocrinol..

[91] Sbaï D., Narcy C., Thompson G.N., Mariotti A., Poggi F., Saudubray J.M., Bresson J.L. (1994). Contribution of odd-chain fatty acid oxidation to propionate production in disorders of propionate metabolism. Am. J. Clin. Nutr..

[92] Gozzo S., Oliverio A., Salvati S., Serlupi-Crescenzi G., Tagliamonte B., Tomassi G. (1982). Effects of dietary phospholipids and odd-chain fatty acids on the behavioural maturation of mice. Food Chem. Toxicol..

[93] Brass E.P., Beyerinck R.A. (1988). Effects of propionate and carnitine on the hepatic oxidation of short- and medium-chain-length fatty acids. Biochem. J..

